# Serum calcium and phosphate levels and carotid atherosclerotic plaque characteristics: a retrospective study by high-resolution MR vessel wall imaging

**DOI:** 10.3389/fneur.2025.1571205

**Published:** 2025-05-02

**Authors:** Xiaowei Song, Hongliang Zhao, Zhuoma Pengmao, Duoduo Hou, Xihai Zhao, Zhuozhao Zheng, Jian Wu

**Affiliations:** ^1^Beijing Tsinghua Changgung Hospital, School of Clinical Medicine, Tsinghua University, Beijing, China; ^2^Center for Biomedical Imaging Research, Department of Biomedical Engineering, School of Medicine, Tsinghua University, Beijing, China; ^3^IDG/McGovern Institute for Brain Research, School of Medicine, Tsinghua University, Beijing, China

**Keywords:** ischemic stroke, atherosclerosis, carotid artery, calcium, phosphate

## Abstract

**Background and aims:**

Serum calcium (Ca), phosphate (P), and calcium-phosphate product (CPP) are associated with cardiovascular disease and atherosclerosis in patients with chronic kidney disease. However, it remains unclear whether this relationship persists in individuals with carotid artery atherosclerosis of acute ischemic stroke. We investigated the association between serum Ca, P, as well as CPP, and carotid artery atherosclerotic plaque in acute ischemic stroke patients.

**Methods and results:**

A total of 251 ischemic stroke participants with carotid artery atherosclerosis (mean age: 68 years; male: 80.1%) were retrospectively enrolled at a comprehensive stroke center. Serum Ca and P levels were obtained from blood tests after admission. Carotid artery plaque burden and vulnerability were evaluated using high-resolution magnetic resonance vessel wall imaging. Subsequently, the associations between serum Ca, P, as well as CPP, and the characteristics of atherosclerotic plaques were analyzed using multivariate linear and logistic regression analyses. Finally, the consistency of these associations was also explored across different subgroups. As a result, serum P and CPP levels were associated with carotid artery plaque burden, presented as maximum wall thickness (max WT), wall area, and lipid-rich necrotic core (LRNC), in univariate analysis, with *β* = −0.205, 95% CI (−0.348, −0.061), *β* = −0.258, 95% CI (−0.405, −0.113), OR = 0.182, 95% CI (0.034, 0.975) for P, and *β* = −0.203, 95% CI (−0.346, −0.059), *β* = −0.221, 95% CI (−0.366, −0.074), OR = 0.466, 95% CI (0.237, 0.915) for CPP, respectively. In multivariate regression analysis, the serum P level was independently associated with wall area, *β* = −0.211, 95% CI (−0.367, −0.052).

**Conclusion:**

Lower serum phosphorus levels are associated with an increased carotid artery plaque wall area.

## Introduction

Previous studies have demonstrated the association between serum calcium/phosphate metabolism and atherosclerosis, as well as a relationship with cardiovascular disease (1). Most studies conducted on patients with coronary artery disease indicated a positive correlation between serum calcium-phosphate levels and coronary artery calcification (2, 3). However, conclusions regarding its association with cerebrovascular atherosclerosis, particularly atherosclerotic plaque vulnerability, have not been established.

Few studies investigating the relationship between serum calcification/phosphate levels and cerebrovascular atherosclerosis have yielded inconsistent results. A Korean retrospective study investigated the association between serum calcium phosphate level and intracranial atherosclerosis in stroke-free patients, and the results suggested that corrected serum calcium concentrations are positively associated with the presence of intracranial atherosclerosis on MRA, but no association was found between uncorrected serum calcium, phosphate, and intracranial atherosclerosis (4). Another study conducted in type 2 diabetes mellitus (T2DM) without kidney disease compared the serum calcium phosphate level in patients with and without subclinical carotid atherosclerosis (defined as presence of carotid plaque on carotid ultrasound imaging), and the results demonstrated that patients with subclinical carotid atherosclerosis have a higher serum phosphate and calcium-phosphate product level than those without subclinical carotid atherosclerosis. After further adjustment for confounders, serum calcium-phosphate product is associated with subclinical carotid atherosclerosis independently (5). In addition, a retrospective study including 1,034 patients with first-ever stroke suggested that serum phosphate level was related to neither intracranial nor extracranial atherosclerosis on DSA (6). However, most of these studies focused on specific populations, such as individuals with chronic kidney disease or diabetes, making it difficult to extrapolate the findings to all stroke patients; and the evaluations of atherosclerosis also varied, as no carotid artery plaque compositions were involved.

The aim of this study was to investigate the association between serum calcium, phosphate, as well as calcium-phosphate product levels, and carotid artery atherosclerotic plaque, both plaque burden and vulnerability, as assessed by high-resolution MR vessel wall imaging (HR MR-VWI).

## Methods

### Study design

Retrospective study.

### Subjects

Ischemic stroke associated with carotid artery atherosclerosis patients admitted to a comprehensive stroke center during 2019–2022 were consecutively screened. The inclusion criteria are as follows: (1) acute ischemic stroke confirmed by brain MRI, (2) symptom onset within 2 weeks, (3) stroke was caused by carotid artery atherosclerosis, and (4) availability of carotid artery plaque MRI vessel wall imaging. Subjects were excluded if they met any of the following criteria: (1) non-atherosclerotic carotid artery lesions, (2) concurrent ipsilateral severe (>50%) intracranial atherosclerosis evaluated on MRA, (3) poor image quality, and (4) parathyroid function disorders.

A total of 251 subjects who met the criteria mentioned above were ultimately included in the study. Subjects were excluded from the analysis for the following reasons: carotid artery occlusion 25, dissection 6, and coexisting severe intracranial stenosis 12.

The study protocol was approved by the local ethics committee, and the requirement for subjects’ consent was waived due to the retrospective analysis. All the data obtained were de-identified to protect patient confidentiality.

### Demographic characteristics and assessment of vascular risk factors

Demographic characteristics and atherosclerotic risk factors—including hypertension, diabetes, hyperlipidemia, coronary heart disease (CAD), and smoking—were obtained from electronic health records.

### Serum calcium and phosphate measurement

Blood samples were collected early in the morning after an overnight fast, with the last meal typically consumed 10 h prior to the blood draw. The samples were analyzed to measure serum calcium, phosphate, albumin, glucose, total cholesterol, high-density lipoprotein (HDL) cholesterol, and triglycerides. Low-density lipoprotein (LDL) cholesterol was estimated using the method described by Friedewald et al. (7). Measurements of serum calcium, phosphate, and albumin concentrations were conducted using standard autoanalyzer techniques (Modular DP analyzer, Roche Diagnostics, Mannheim, Germany). The serum calcium concentration was corrected for serum albumin concentration using the following formula: corrected calcium (mmol/L) = measured total calcium (mmol/)- [0.02 × serum albumin(g/L)] + 0.8. All the blood samples were processed in the same lab with the same protocol.

### Carotid artery MR vessel wall imaging

Magnetic resonance imaging was conducted using a Philips 3.0 T MR scanner (Achieva TX, Philips Healthcare, Best, the Netherlands) equipped with a custom-designed 36-channel neurovascular coil. The MR imaging parameters were as follows: 3D MERGE: fast field echo (FFE), repetition time (TR)/echo time (TE) 9.2/4.3 msec, flip angle 6°, field of view (FOV) 4 × 16 × 25 cm^3^, and spatial resolution of 0.8 × 0.8 × 0.8 mm^3^; T2-VISTA: turbo spin echo, TR/TE 2500/278 msec, flip angle 90°, FOV 4 × 16 × 25 cm^3^, and spatial resolution 0.8 × 0.8 × 0.8 mm^3^; Simultaneous Non-contrast Angiography and intraPlaque Hemorrhage (SNAP): FFE, TR/TE 9.9/4.8 msec, flip angle 11/5°, FOV 4 × 16 × 25 cm^3^, and spatial resolution 0.8 × 0.8 × 0.8 mm^3^.

### Image interpretation

Carotid artery MR vessel wall images were analyzed by two reviewers with >5 years experience in neurovascular imaging, using an MR workstation (Extended MR Work Space 2.6.3.4, Best, the Netherlands). The reviewers worked independently, and in cases of inconsistency, a third senior radiologist was involved to reach a consensus. The presence or absence of atherosclerotic plaque at each carotid artery was determined; the atherosclerotic plaque is defined as the eccentric thickening of the vessel wall. The degree of arterial luminal stenosis was measured using the NASCET criteria (8). The carotid artery plaque burden index included maximum carotid wall thickness (max WT), which is the maximum segmental wall thickness of 12 segments measured at the slice with the largest lipid core area, or the maximum segmental wall thickness if no core was present; wall area; normalized wall index (wall area /[lumen area + wall area]), and degree of stenosis. The carotid artery plaque stability index included intraplaque hemorrhage (IPH), defined as the presence of IPH in each plaque when hyperintensity on SNAP images exhibited a signal intensity ≥1.5 times that of adjacent muscle or cerebral parenchyma; calcification; lipid-rich necrotic core (LRNC); thinned ruptured fibrous cap (TRFC); and irregular surface (9). The consistency test for image interpretation between the two readers was good, with kappa = 1.0 (*p* < 0.01) for diagnosing IPH and an ICC = 0.94 (95% CI: 0.85–0.97) for determining the max WT measurements.

### Statistical analysis

Continuous variables with a normal distribution were described as mean ± standard deviation. T-tests were used for comparisons between groups, while categorical variables were presented as numbers (percentages), with chi-square tests used for group comparisons. Linear regression was utilized to assess the association between calcium and phosphate levels and plaque burden (max WT and wall area). Logistic regression was applied to identify the association between calcium and phosphate levels and atherosclerotic plaque vulnerability (IPH, calcification, TRFC, and LNRC). In the multivariate regression analysis, we adjusted for demographic characteristics and risk factors step by step. All *p*-values were set at 0.05, and all statistical analyses were performed using SPSS version 22.0 (IBM, New York, USA). Subgroup analysis forest plots were generated using Stata 17.0.

## Result

### Basic characteristic of subjects included

A total of 251 acute ischemic stroke patients with carotid atherosclerosis were included in the study, with a mean age of 68 years. Of these patients, 80.1% were male. The prevalence of coexisting hypertension, diabetes, and hyperlipidemia was 75.2, 41.7, and 50.8%, respectively. The mean serum calcium and phosphorus levels were 2.26 ± 0.11 mmol/L and 1.16 ± 0.19 mmol/L, retrospectively (see Table 1).

**Table 1:** Basic characteristics of subjects included.

Variables, M ± SD or *n* (%)	Total (*N* = 251)
Age (years)	68 ± 10
Male	205 (80.1)
Hypertension	191 (75.2)
Diabetes	105 (41.7)
Hyperlipidemia	129 (50.8)
CAD	58 (23.1)
Smoking	174 (69.6)
Hcy (umol/L)	17.4 ± 8.3
SBP (mmHg)	145 ± 20
DBP (mmHg)	78 ± 13
Glucose (mmol/L)	6.12 ± 2.00
TC (mmol/L)	4.03 ± 0.94
TG (mmol/L)	1.46 ± 0.78
LDL-C(mmol/L)	2.53 ± 0.94
HDL-C(mmol/L)	0.98 ± 0.25
eGFR(ml/min/1.73m^2^)	86.53 ± 19.63
Ca(mmol/L)	2.26 ± 0.11
P(mmol/L)	1.16 ± 0.19
Ca*P	2.63 ± 0.46

### Carotid artery plaque burden and vulnerability in subjects included

Carotid artery plaque characteristics are presented in Table 2. Among the included patients, 53.0% exhibited less than 50% stenosis, with a mean max WT of 4.29 mm. Additionally, 42.0% of the patients had IPH, while 39.4% showed atherosclerotic plaque calcification. Furthermore, 35.5% of the patients exhibited LRNC and TRFC characteristics, respectively.

**Table 2:** Carotid atherosclerotic plaque burden and vulnerability.

Carotid atherosclerotic plaque characteristics M ± SD or *n* (%)	Total (*n* = 251)	95% CI
Stenosis		
≤30%	92 (36.8)	
30–49%	41 (16.3)	
50–69%	44 (17.5)	
70–99%	74 (29.4)	
Max WT (mm)	4.29 ± 1.45	
Lumen area(mm^2^)	0.22 ± 0.15	
Wall area (mm^2^)	0.50 ± 0.19	
NWI	0.70 ± 0.13	
Eccentricity index	0.66 ± 0.14	
IPH	97(42.0)	35.5,48.1
Calcification	91 (39.4)	32.9,45.9
Irregular surface	73 (31.6)	26.0, 38.1
LRNC	82 (35.5)	29.4, 41.6
TRFC	82 (35.5)	29.4, 41.6

### Association between Ca, P with carotid artery plaque burden and vulnerability

Table 3 demonstrates the association between serum Ca, P, as well as CPP and atherosclerotic plaque characteristics in a univariate analysis. Both P and CPP were correlated with max WT, wall area, and LRNC in univariate regression. However, no positive associations between Ca and atherosclerotic plaque features were detected in this analysis.

**Table 3:** Association between serum calcium phosphate and carotid atherosclerotic plaque characteristics in univariate analysis.

	Max WT			Wall area			NWI			Eccentricity index			IPH			Calcification			TRFC			LRNC		
	β	95%CI	*p*	β	95% CI	*p*	β	95%CI	*p*	β	95% CI	*p*	OR	95% CI	*p*	OR	95%CI	*p*	OR	95%CI	*p*	OR	95%CI	*p*
Ca	−0.073	−2.742, 0.826	0.291	0.001	−0.144, 0.145	0.994	−0.082	−0.227, 0.059	0.248	−0.016	−0.160, 0.127	0.820	2.020	0.208, 19.657	0.545	0.672	0.066, 6.873	0.738	1.969	0.169, 22.866	0.588	0.214	0.018, 2.509	0.219
P	−0.205	−0.348, −0.061	0.006	−0.258	−0.405, −0.113	0.001	−0.044	−0.190, 0.105	0.568	−0.128	−0.281, 0.020	0.090	0.415	0.092, 1.881	0.254	0.307	0.064, 1.476	0.140	0.396	0.078, 2.002	0.263	0.182	0.034, 0.975	0.047
Adjusted Ca*	−0.080	−0.218, 0.057	0.247	−0.011	−0.149, 0.128	0.881	−0.055	−0.191, 0.084	0.443	−0.068	−0.208, 0.070	0.327	0.859	0.061, 12.103	0.911	0.779	0.051, 11.860	0.857	0.669	0.036, 12.448	0.788	0.044	0.001, 1.831	0.101
Ca*P	−0.203	−0.346, −0.059	0.006	−0.221	−0.366, −0.074	0.003	−0.070	−0.214, 0.078	0.360	−0.123	−0.275, 0.026	0.103	0.733	0.401, 1.340	0.313	0.638	0.341, 1.194	0.160	0.733	0.385, 1.395	0.345	0.466	0.237, 0.915	0.027

^*^Adjusted Ca: albumin-corrected calcium.

### Association between serum P, CPP, and plaque characteristics in multivariate regression

In multivariate regression, after adjusting for age and sex in Model 1, as well as cardiovascular risk factors in Model 2, serum P is independently associated with wall area, with *β* = −0.211, 95% CI (−0.367, −0.052). Additionally, CPP is marginally correlated with wall area, with β = −0.157, 95% CI (−0.314, 0.004), *p* = 0.056 (see Table 4).

**Table 4:** Association between serum P, CPP levels, and carotid artery atherosclerotic plaque in multivariate regression.

	Max WT						Wall area						LRNC					
	Model 1			Model 2			Model 1			Model 2			Model 1			Model 2		
	β	95% CI	*p*	β	95% CI	*p*	β	95% CI	*p*	β	95% CI	*p*	OR	95% CI	*p*	OR	95% CI	*p*
P	−0.115	−0.264, 0.035	0.131	−0.134	−0.286, 0.021	0.091	−0.190	−0.344, −0.036	0.016	−0.211	−0.367, −0.052	0.010	0.309	0.052, 1.843	0.198	0.249	0.040, 1.563	0.128
Ca*P	−0.112	−0.261, 0.037	0.141	−0.128	−0.280, 0.028	0.108	−0.147	−0.300, 0.008	0.063	−0.157	−0.314, 0.004	0.056	0.572	0.280, 1.171	0.126	0.532	0.254, 1.114	0.094

Adjust age and sex in model 1. Adjust age, sex, hypertension, diabetes, hyperlipidemia, smoking, and eGFR in model 2.

### Association between serum P, CPP, and carotid artery atherosclerosis in different subgroups

**Figure 1:**
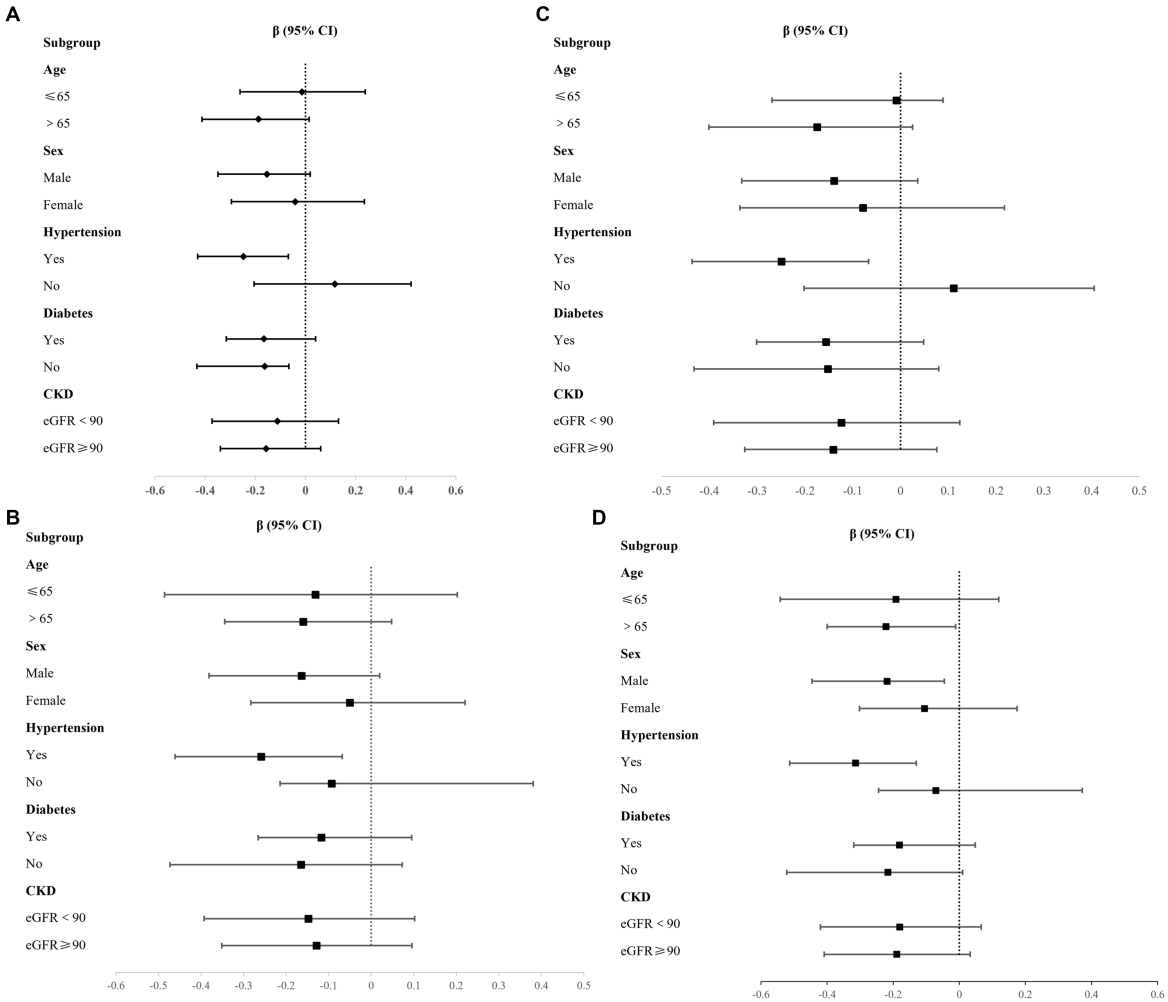
Association between P, CPP and plaque burden in different subgroups. **(A)** P and Max WT; **(B)** P and Wall area; **(C)** CPP and Max WT; **(D)** CPP and Wall area.

We explored the relationship between P, CPP, and carotid atherosclerotic plaque characteristics across various subgroups, including age, sex, hypertension, diabetes, and CKD subgroups (Supplementary Tables 1, 2). After adjusted cofounding, the results indicated that P is associated with max WT in hypertensive subgroups, *β* = −0.248, 95% CI (−0.430, −0.068) (Figure 1A), also associated with wall area in individuals over 65 years of age(*β* = −0.222, 95% CI [−0.400, −0.011]), male participants (*β* = −0.219, 95% CI [−0.446, −0.045]), and those with hypertension (*β* = −0.314, 95% CI [−0.513, −0.130]) (Figure 1B). Additionally, the positive relationship between CPP and carotid artery plaque burden (max WT and wall area) can still be established in the hypertensive subgroup, with *β* = −0.249, 95% CI (−0.436, −0.067), and *β* = −0.259, 95% CI (−0.461, −0.068) (Figures 1C,D). No other significant differences were identified in the remaining subgroups.

## Discussion

This study investigated the association between serum calcium phosphate levels and carotid atherosclerosis, assessed comprehensively through HR-MR VWI, focusing on atherosclerotic plaque burden and vulnerability. The results demonstrated a positive relationship between serum phosphate levels and carotid atherosclerosis, as indicated by wall area, after adjusting for confounding factors. CPP was associated with the wall area marginally. Further subgroup analysis suggested potential differences in this correlation based on age, sex, and hypertension status. The results of this study could provide some insight into serum mineral metabolism and carotid atherosclerosis.

### Serum phosphate level and atherosclerosis

Serum phosphate has been established as a risk factor for cardiac calcification, cardiovascular disease, and mortality in populations with chronic kidney disease (10), has also been recognized as a risk factor for ischemic stroke in hemodialysis patients (11, 12). Additionally, some studies suggest that phosphate levels within the conventional normal range may also contribute to cardiovascular disease (10). A meta-analysis of the general population indicated that elevated serum phosphate is associated with cardiovascular mortality and subclinical coronary atherosclerosis (13). However, data about serum phosphate and carotid artery atherosclerosis were limited. In a study of 1,034 patients with first-ever acute cerebral infarction, serum phosphate was associated with neither intracranial nor extracranial atherosclerosis (6). Another cross-sectional study in the general population demonstrated that a high phosphate level is correlated with cardiovascular disease, while a lower phosphate level is correlated with metabolic syndrome (14). Our study did find the association between serum phosphate level and carotid atherosclerosis after adjusting for confounding, especially max WT, wall area, and LRNC. This finding is in contrast to the results in the coronary artery. The inconsistency can be explained by the population included, the vascular bed heterogeneity, as well as the atherosclerosis definitions. In this study, we included acute ischemic stroke patients with carotid artery atherosclerosis, and we used HR-MR VWI to quantitatively assess carotid atherosclerosis, which is more sensitive than intracranial artery stenosis on MRA. While previous study on coronary artery disease usually evaluates atherosclerosis on CTA. The mechanism of low serum phosphate contributes to atherosclerosis could be caused by glucose metabolism or malnutrition–inflammation complex syndrome (12). As we know, phosphate is a component of cell membranes and plays an important role in mediating intracellular signaling, so lower serum phosphate levels may have adverse implications for vascular biology and may affect several organ systems (15). There is one study validated that low serum phosphate level is associated with glucose tolerance, insulin sensitivity in non-diabetic subjects (16). While we did not find a significant difference in P and carotid atherosclerosis in the diabetes and non-diabetes subgroups due to the limited sample size. From a pathophysiological perspective, animal experiments demonstrated that excess phosphate intake decreased atherosclerosis formation partly by changing the profile of peripheral monocytes or inducing apoptosis of macrophages in apolipoprotein E-deficient mice (17). However, our exploratory findings could not validate the causality; more longitudinal studies are needed in the future.

We also find that the positive associations between serum phosphate and carotid artery atherosclerosis differ in different sex groups; the main results can be established in males but not in females. A previous study regarding phosphate and subclinical carotid atherosclerosis also suggested the sex difference (18). As for the causes of this result, there are more male patients included in our study, which could result in some population bias; Second, age and associated hormone levels could also affect the result. Therefore, more balanced data, including different age and sex groups, are needed to validate the results.

### Ca-P product and atherosclerosis

The relationship between CPP and atherosclerosis or cardiovascular disease has not been concluded. Data from Atherosclerosis Risk in Communities (ARIC) study suggested that the calcium-phosphate product is associated with stroke in a prospective cohort (HR = 1.15, 95% CI 1.05–10.26, *p* = 0.0017) (19). Association between CPP and coronary atherosclerosis assessed by cardiac computed tomography angiography had been established in a cohort of 7,553 participants with normal kidney function, especially with the presence of calcified coronary atherosclerotic plaque (3). As for CPP and subclinical carotid atherosclerosis, a study of 303 subjects was conducted in patients with type 2 diabetes mellitus (T2DM) without kidney disease or previous cardiovascular disease, clinical variables and carotid ultrasound imaging were obtained. Subclinical atherosclerosis was defined as the presence of carotid artery atherosclerotic plaques (main study outcome), and the result suggested that CPP was positively associated with the presence of carotid plaques (OR(adj) = 1.078; 95% CI: 1.017–1.142) (5). Our result demonstrated the association between CPP and max WT, wall area, and LRNC in univariate analysis, but with limited significance in multivariate analysis. The possible reasons for this result could be contributed to the population included, sample size, and some cofounding factors, a prospective study with larger sample size is needed to validate the result in the future.

### Significance and limitations

To our best knowledge, this is the first study to investigate serum calcification-phosphate metabolism and carotid artery atherosclerosis quantitatively on HR-MR VWI. The results of our study could provide some insights about serum mineral levels and carotid artery atherosclerosis in acute ischemic stroke, as well as some potential differences in different subgroups.

There are some innovations of our study. First, we use HR-MR VWI to quantitatively evaluate atherosclerotic plaque burden and vulnerability, which is different from previous calcification scores on CCTA. Second, we investigate the calcium phosphate levels and carotid artery atherosclerosis in all acute ischemic stroke patients, but not diabetes or chronic kidney disease. The results of our study could be helpful in the exploration of the potential risk factors of carotid artery atherosclerosis, also provide some evidence for future mechanism studies.

There were some limitations about this study, first, the population in this study is limited to a single center in China, with a male-dominated cohort. This limits the generalizability of findings to other populations, particularly women and non-Asian ethnic groups. A larger population with more balanced sex distribution is still needed in the future. Second, the cross-sectional analysis fails to validate the causal relationship between serum mineral levels and atherosclerosis. Finally, no other dietary intake of calcium phosphate, as well as vitamin D levels, were included in this study. More details of factors in calcium and phosphorus metabolism will be helpful in the mechanism explanation. Further study, including serum calcium phosphate intake and Vitamin D supplement, will be needed in the future.

## Conclusion

Serum phosphate was independently associated with carotid atherosclerotic plaque burden in acute ischemic stroke. And this correlation could be significant in males, older age, and hypertension subgroups.

## Data Availability

The original contributions presented in the study are included in the article/Supplementary material, further inquiries can be directed to the corresponding author.
