# Supplementary material for: Serum calcium and phosphate levels and carotid atherosclerotic plaque characteristics: a retrospective study by high-resolution MR vessel wall imaging
Source: Front Neurol. 2025 May 2;16:1571205. doi: 10.3389/fneur.2025.1571205 (PMC12081255; doi:10.3389/fneur.2025.1571205)
Supplement: Supplementary file 1 [file Table_1.docx]

**Supplementary Tables**

**S-Table 1 Association between P and carotid artery plaque burden and vulnerability**

|  | **Max WT** |  |  | **Wall area** |  |  | **LNRC** |  |  |
| --- | --- | --- | --- | --- | --- | --- | --- | --- | --- |
|  | **β** | **95%CI** | **P** | **β** | **95%CI** | **P** | **OR** | **95%CI** | **P** |
| **Age (years)** |  |  |  |  |  |  |  |  |  |
| **≤65** | -0.014 | -0.261，0.238 | 0.927 | -0.192 | -0.542，0.119 | 0.205 | 0.756 | 0.034，16.971 | 0.860 |
| **>65** | -0.187 | -0.413，0.014 | 0.066 | -0.222 | -0.400，-0.011 | 0.039 | 0.157 | 0.013，1.907 | 0.146 |
| **Sex** |  |  |  |  |  |  |  |  |  |
| **Male** | -0.154 | -0.349，0.019 | 0.079 | -0.219 | -0.446，-0.045 | 0.017 | 0.168 | 0.022，1.289 | 0.086 |
| **Female** | -0.041 | -0.295，0.235 | 0.817 | -0.105 | -0.302，0.175 | 0.589 | 0.191 | 0.000，610.736 | 0.688 |
| **Hypertension** |  |  |  |  |  |  |  |  |  |
| **Yes** | -0.248 | -0.430，-0.068 | 0.007 | -0.314 | -0.513，-0.130 | 0.001 | 0.118 | 0.012，1.163 | 0.067 |
| **No** | 0.117 | -0.205，0.421 | 0.489 | -0.071 | -0.244，0.372 | 0.676 | 0.431 | 0.008，22.569 | 0.677 |
| **Diabetes** |  |  |  |  |  |  |  |  |  |
| **Yes** | -0.166 | -0.316，0.040 | 0.126 | -0.181 | -0.319，0.048 | 0.144 | 0.363 | 0.021，6.352 | 0.488 |
| **No** | -0.163 | -0.433，-0.066 | 0.148 | -0.216 | -0.522，0.010 | 0.059 | 0.104 | 0.007，1.513 | 0.098 |
| **CKD** |  |  |  |  |  |  |  |  |  |
| **eGFR＜90** | -0.112 | -0.373，0.132 | 0.345 | -0.180 | -0.420，0.066 | 0.151 | 0.075 | 0.004，1.421 | 0.084 |
| **≥90** | -0.157 | -0.340，0.061 | 0.171 | -0.189 | -0.409，0.033 | 0.095 | 0.671 | 0.046，9.739 | 0.770 |

**S-Table 2 Association between CPP and carotid artery plaque burden and vulnerability**

|  | **Max WT** |  |  | **Wall area** |  |  | **LRNC** |  |  |
| --- | --- | --- | --- | --- | --- | --- | --- | --- | --- |
|  | **β** | **95% CI** | **P** | **β** | **95% CI** | **P** | **OR** | **95% CI** | **P** |
| **Age** |  |  |  |  |  |  |  |  |  |
| **≤65** | -0.009 | -0.269，0.089 | 0.953 | -0.131 | -0.486，0.202 | 0.411 | 0.729 | 0.198，2.684 | 0.635 |
| **＞65岁** | -0.175 | -0.401，0.025 | 0.083 | -0.160 | -0.344，0.048 | 0.137 | 0.490 | 0.183，1.312 | 0.156 |
| **Sex** |  |  |  |  |  |  |  |  |  |
| **Male** | -0.139 | -0.332，0.036 | 0.115 | -0.164 | -0.381，0.020 | 0.077 | 0.468 | 0.207，1.060 | 0.069 |
| **Female** | -0.079 | -0.336，0.217 | 0.662 | -0.050 | -0.283，0.221 | 0.801 | 0.351 | 0.011，11.492 | 0.556 |
| **Hypertension** |  |  |  |  |  |  |  |  |  |
| **Yes** | -0.249 | -0.436，-0.067 | 0.008 | -0.259 | -0.461，-0.068 | 0.009 | 0.425 | 0.168，1.080 | 0.072 |
| **No** | 0.111 | -0.202，0.405 | 0.503 | -0.093 | -0.214，0.381 | 0.572 | 0.532 | 0.112，2.518 | 0.426 |
| **Diabetes** |  |  |  |  |  |  |  |  |  |
| **Yes** | -0.156 | -0.301，0.048 | 0.152 | -0.117 | -0.266，0.095 | 0.349 | 0.541 | 0.169，1.727 | 0.299 |
| **No** | -0.152 | -0.432，0.080 | 0.176 | -0.165 | -0.473，0.073 | 0.148 | 0.450 | 0.152，1.335 | 0.150 |
| **CKD** |  |  |  |  |  |  |  |  |  |
| **eGFR＜90** | -0.124 | -0.391，0.124 | 0.304 | -0.148 | -0.393，0.102 | 0.244 | 0.312 | 0.092，1.066 | 0.063 |
| **≥90** | -0.141 | -0.326，0.076 | 0.219 | -0.129 | -0.351，0.096 | 0.260 | 0.849 | 0.291，2.476 | 0.764 |
